# Ballistic Impact Behaviour of Glass/Epoxy Composite Laminates Embedded with Shape Memory Alloy (SMA) Wires

**DOI:** 10.3390/molecules26010138

**Published:** 2020-12-30

**Authors:** Luv Verma, Jefferson Andrew, Srinivasan M. Sivakumar, Gurusamy Balaganesan, Srikanth Vedantam, Hom N. Dhakal

**Affiliations:** 1 Department of Applied Mechanics, IIT Madras, Chennai 600036, India; luvverma2011@gmail.com (L.V.); mssiva@iitm.ac.in (S.M.S.); 2 Department of Mechanical Engineering, Khalifa University, Abu Dhabi 127788, UAE; 3 Department of Mechanical Engineering, IIT Jammu, Jammu 181221, India; balaganesan.gurusamy@iitjammu.ac; 4 Department of Engineering Design, IIT Madras, Chennai 600036, India; srikanth@iitm.ac.in; 5 School of Mechanical and Design Engineering, University of Portsmouth, Portsmouth PO1 3DJ, UK

**Keywords:** superelastic shape memory alloy (SE-SMA) wires, impact, GFRP, glass/epoxy composite materials, damage mechanisms

## Abstract

This paper aims to estimate the enhancement in the energy absorption characteristics of the glass fiber reinforced composites (GFRP) by embedding prestrained pseudo-elastic shape memory alloy (SMA) that was used as a secondary reinforcement. The pseudo-elastic SMA (PE-SMA) embedded were in the form of wires and have an equiatomic composition (i.e., 50%–50%) of nickel (Ni) and titanium (Ti). These specimens are fabricated using a vacuum-assisted resin infusion process. The estimation is done for the GFRP and SMA/GFRP specimens at four different impact velocities (65, 75, 85, and 103 m/s) using a gas-gun impact set-up. At all different impact velocities, the failure modes change as we switch from GFRP to SMA/GFRP specimen. In the SMA/GFRP specimen, the failure mode changed from delamination in the primary region to SMA-pull out and SMA deformation. This leads to an increase in the ballistic limit. It is observed that energy absorbed by SMA/GFRP specimens is higher than the GFRP specimens subjected to the same levels of impact energy. To understand the damping capabilities of SMA embedment, vibration signals are captured, and the damping ratio is calculated. SMA dampens the vibrations imparted by the projectile to the specimen. The damping ratio of the SMA/GFRP specimens is higher than the GFRP specimens. The damping effect is more prominent below the ballistic limit when the projectile got rebounded (65 m/s).

## 1. Introduction

In the past few decades, research has established the prowess of composite materials over conventional metals due to their high stiffness and strength to weight ratios [1]. They have outmatched the metals when it comes to their applications, where stress dominates in specific directions of the structural element. This helps afford strength reductions along with non-dominant directions. These composites that render the above advantage should also be equally resistant to impact conditions that may occur in the lifetime of the components they are used in. Assessment of performance of the composites during those impact events and quantification of energy dissipation will help the design of composites better. Early studies on the impact behavior of glass and carbon fiber based composite materials point to their low energy absorption capabilities as these primary reinforcement fibers are brittle [1,2,3,4]. To raise their standards under impact conditions, it is important to increase their energy dissipation capabilities. One of the ways of increasing energy absorption is to embed composites with high ductile materials [5]. One such material that has good ductility is the shape memory alloys (pseudo-elastic shape memory alloy: PE-SMA) [6,7].

PE-SMAs are highly ductile alloys with high endurance in the plastic deformation regime. This behavior of PE-SMA is attributed to their phase transformation mechanisms that occur internally due to the thermomechanical loading [8,9]. Thus, SMA embedded composite (also called the SMA hybrid composite) can serve well in the applications where resistance to penetration and damage during ballistic impact plays an important role (examples: helmets, car bumpers, bird hits on wings, and ballistic armor protection) [10,11]. Early studies on SMA hybrid graphite composite specimens reveal a significant increase in the absorbed energy and peak impact force at low-velocity impacts [5]. Numerical and experimental investigations were also carried out on these SMA hybrid composite (SMAHC) plates confirming an increase in the absorbed energy under low-velocity impact [12]. To enhance the ductility, studies have been conducted under low-velocity impact to evaluate the dependence of SMAHC on SMA prestrain, its volume fraction, their orientation, and their position within the laminates. Researchers have seen improvements in the vibration and damping properties by controlling SMA prestrain during specimen preparation [13,14,15]. Prestraining SMA in both directions embedded in composite plates is found to reduce deflections and stresses at higher temperatures [16]. However, the effect of laying bidirectional SMA in graphite-epoxy composites showed a negative effect leading to an increase in damage area in comparison with specimens with unidirectional SMA [10]. On the other hand, SMA stitched to the glass fiber reinforced composites (GFRP) helps to decrease the number of translaminar cracks and increase the tensile strength [17,18].

High-velocity impact experiments were conducted on SMAHC [19,20], and impact zones were observed visually. They showed more localized impact perforations than in the low-velocity regimes, indicating different modes of failure at different ranges of velocities. While low and high-velocity regimes tests are available in the open literature [21,22], scant information is available on the performance of pseudo-elastic SMA composites in the range of medium velocity impact. At low velocities, the projectile will not be able to penetrate and at higher velocities, it passes through the wires by pushing them aside. Change in the failure modes changes the process and the amount of energy absorption [23,24]. Given that the failure modes are different at low and high velocities, failure modes at medium velocities cannot be understood without conducting experiments at such velocities.

Embedded pseudo-elastic SMA also functions as a damper [25,26,27]. It reduces the vibration generated in the specimen while undergoing the impact loads. Damping in the material during the impact at regions away from the impact is also an important consideration to protect against damage due to excessive vibration in those regions. Testing for such damping effects with SMA embedment also needs attention. There is a lack of literature on the damping effect of pseudo-elastic SMA based composite specimens.

Therefore, in this work, the performance of pseudo-elastic SMA embedded composites under medium velocity impacts (65, 75, 85, and 103 m/s) is studied. Additionally, to understand the damping capabilities of the SMA embedded composites, experiments were conducted, and vibration signals were captured at several locations of the specimen for different impact velocities. The results can lead to an assessment of the impact velocity dependent damping ratio of these composites.

## 2. Experimental Procedure

### 2.1. Materials and Fabrication

**Figure 1:**
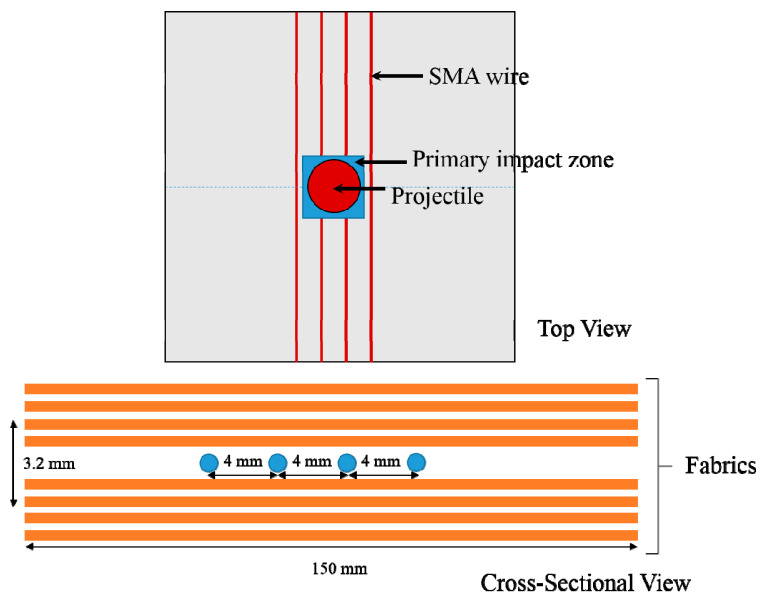
Top view and cross-sectional view of the shape memory alloy (SMA)/glass fiber reinforced composites (GFRP) composite laminates.

Woven roving mat (WRM) glass fiber of areal density 360 g/m^2^ was used as primary reinforcement and pseudo-elastic shape memory alloy (PE-SMA) was used as a secondary reinforcement. The PE-SMA embedded were in the form of wires and have an equiatomic composition (i.e., 50%–50%) of nickel and titanium. Epoxy resin (LY556) and hardener (HY951) were used as matrix material in the weight ratio of 10:1 [28,29]. Table 1, Table 2 and Table 3 summarize the properties of PE-SMA, glass fiber, and epoxy, respectively. Specimen configurations and geometry are represented in Figure 1.

The specimens are prepared in two configurations. In configuration 1, GFRP is prepared by using glass fabrics of eight layers. In configuration 2, the SMA is embedded in between the fourth and fifth layers, as shown in Figure 1. The SMA wires are laid at a distance of 4 mm to each other in the primary impact region. The SMA wires were prestrained to a force of 110 N (which exceeds the phase transformation start stress (Section 3.2. SMA/Epoxy Pull-Out Test). This is helpful in making sure the dissipation characteristics of SMA kick-in early stages of the impact event. The specimens are prepared by the resin infusion process and the size of the laminate is 150 mm × 150 mm. The laminates were allowed to cure at ambient temperature (30 °C) for 24 h under the vacuum pressure of 30 mm Hg. The volume fraction of SMA considered in this study was 0.7% and GFRP fibers were 57%. The wt % of SMA considered in this study was 1% of the fiber weight. The nominal thickness of the laminate was 3.2 ± 0.05 mm.

### 2.2. Quasistatic Tensile Test and SMA Pull-Out Test

**Figure 2:**
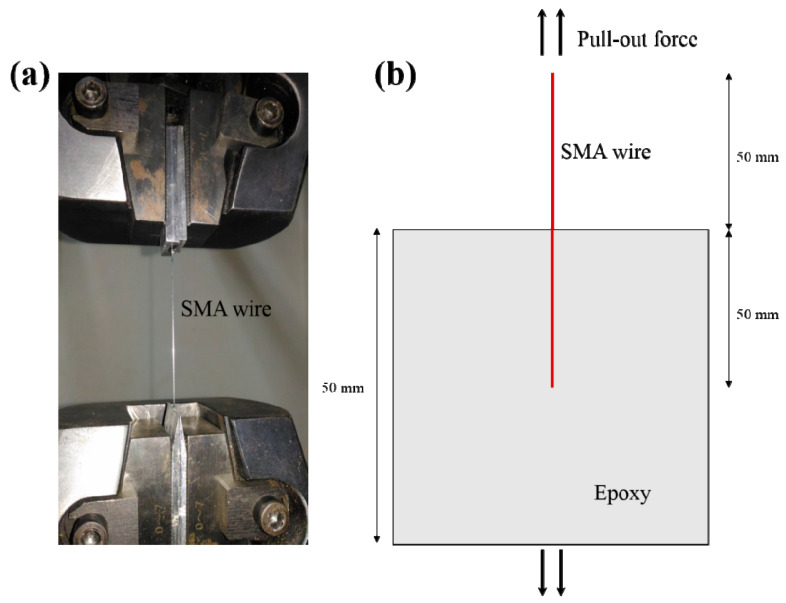
Quasistatic tensile test (**a**) individual SMA wire and (**b**) SMA/epoxy pull-out test.

INSTRON 8801 universal testing machine (UTM, IIT Madras, Chennai, India) of capacity 100 kN was used to perform quasistatic tensile tests (Figure 2a), according to the ASTM standards D3039/3039M [30]. The tensile test has been conducted at a cross-head speed of 1 mm/min. SMA pull-out test (Figure 2b) was conducted to find out the SMA/epoxy bond strength. This fixture of length 25 mm was made with two silicon rubber pieces fixed at the ends to hold the epoxy filled inside the fixture. The SMA was embedded through the hole in the first silicon rubber and extended until the second silicon rubber to keep its position fixed. After filling it with epoxy, a rectangular plate was used to cover the fixture so that the epoxy does not experience any compressive forces while clamping in UTM. At one of the end, the SMA wire was gripped, and at the other end, the rectangular fixture was gripped. The set-up was designed to avoid compressive force on epoxy due to the rectangular fixture. The amount of energy dissipation was found using the load vs. deflection curve obtained before the SMA/epoxy bond failure.

### 2.3. Gas-Gun Impact Test

**Figure 3:**
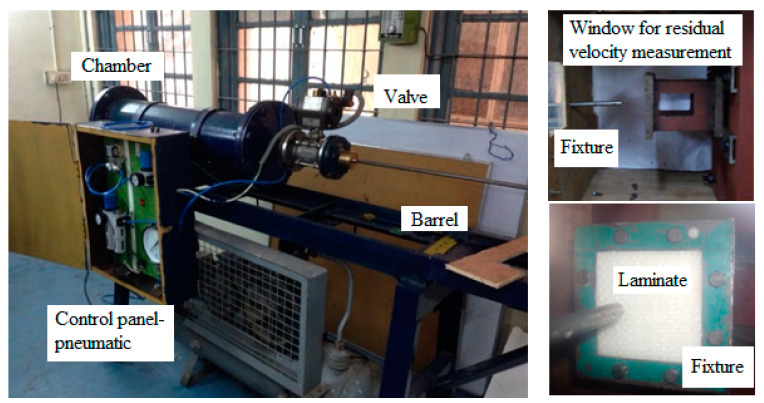
Gas gun experimental set-up used for impact testing on the composite laminates.

Impact tests were done on the specimens using a gas-gun impact set-up, as shown in Figure 3. The specimens were impacted at four different velocities (65, 75, 85, and 103 m/s). A spherical nose steel projectile of diameter 9.5 mm and mass 9 g was used as an impactor. The laminates were clamped on all four sides. A high-speed camera (Phantom V611 at 50,000 fps, IIT Madras, Chennai, India) was placed in a transverse direction to the fixture to capture the projectile velocity before and after the impact (to calculate the energy absorbed by the specimen). As shown in Figure 1, the impact was done in the primary region to understand the behavior of the SMA embedded composites in different velocity ranges. For each testing condition, four specimens were tested.

### 2.4. Vibration Test

Vibration signals have been captured while performing the impact test. A shock accelerometer of capacity 100 kg was used to capture the time response signal through a data acquisition (DAQ) card (NI-PXI 4472), and the response is recorded on a computer. The accelerometer was fixed at a distance ¼ th length of diagonal from one of its corners, which is also a non-nodal line. The time signal is processed with FFT to get the frequency response.

## 3. Results and Discussions

### 3.1. Quasistatic Tensile Response of Individual SMA Wire

**Figure 4:**
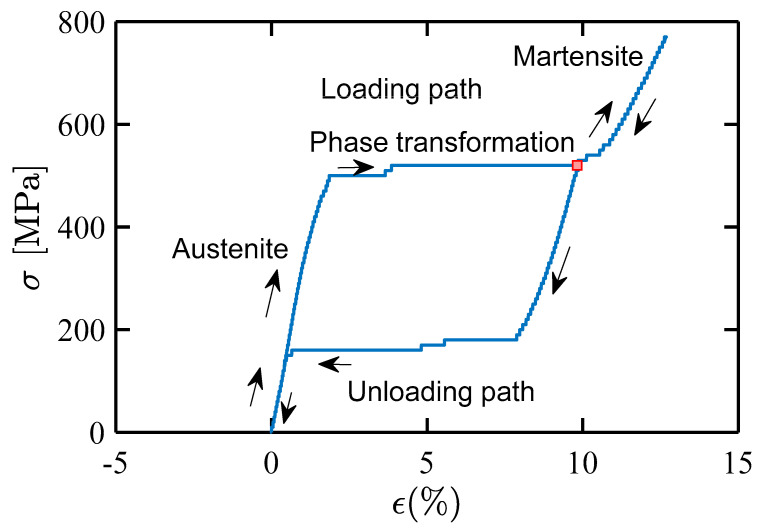
Stress-strain curve of individual SMA wire under quasistatic tensile loading.

The SMA wire in the impact laminates undergoes strain in the longitudinal direction when the projectile strikes. Therefore, a quasistatic test has been done to get the SMA response under tensile loading. Figure 4 shows the stress-strain curve of the SMA wire obtained from the tension test. As the behavior of SMA is known, energy dissipation can be calculated from the area under the curve. An assumption is made that the quasistatic behavior is close to the dynamic behavior of SMA. The reason behind this assumption is that the stress-strain response curve area under dynamic loading is close as that under the quasistatic loading [31]. This implies that the effect on differences in the energy dissipation might be negligible w.r.t the rate of loading.

The initial elastic phase is an austenite phase (Figure 4). After the austenite phase, the phase transformations from austenite to martensite started and completed at almost a constant stress at a point marked by the red dot. This region is where most of the energy dissipation takes place. It is followed by a martensite phase. While unloading, the wire was able to come back to its initial state of zero strain. To achieve visible plastic strains in SMA wire, one has to go above the strain of more than 12%, as shown in the SMA stress-strain curve.

### 3.2. SMA/Epoxy Pull-Out Test

**Figure 5:**
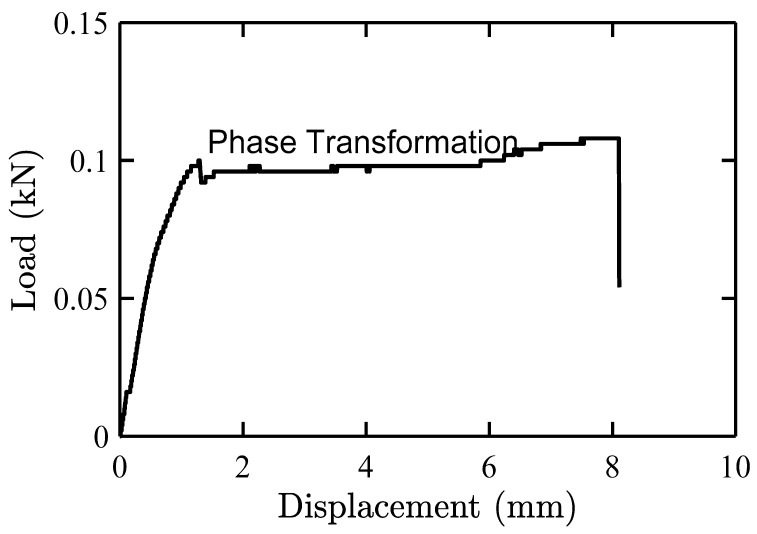
Load vs. displacement curve for SMA/epoxy composite specimen under the pull-out test.

The load vs. displacement curve of SMA/epoxy specimen under the pull-out tensile test is shown in Figure 5. The experiment aims to determine the bond strength between the SMA and epoxy. In the experiment, the deformation of SMA wire before pull-out is observed as the region of SMA phase transformation is visible in the curve. Initially, as the load was applied, the SMA/epoxy system worked together as a single unit until the point where the bond between the two breaks (because the tensile forces being transmitted as the shear forces, acting between SMA and epoxy). At the point where the bond fails, strength goes down as shown in the Figure 5. From the curve, it can be visualized that the load started increasing again starting at the displacement of 6 mm, before which the load was approximately constant. This behavior was exhibited by SMA (Figure 4) while undergoing martensitic loading after phase transformation from martensite to austenite is complete. Therefore, it can be inferred that before the strength of the bond degraded significantly (at 8 mm displacement) and SMA started pulling out of epoxy, the martensitic phase transformation was complete.

From Figure 5, the pull-out strength of SMA from the 25 mm of epoxy mold is given as 0.1 kN, after which the SMA starts slipping out of the epoxy. In the impact specimens, the total length embedded per wire is 150 mm in epoxy, which is approximately 6 times more than that embedded in the pull-out experiment. From the literature it is known that the pull-out load vs. crack length at the interface has a linear relationship and crack length is directly proportional to the embedded fiber length [32]. As a result, it can be inferred that the total pull-out force generated for 150 mm embedded SMA would have an approximate value of 0.6 kN. Therefore, when SMA is prestrained with 0.1 kN of force, as this force is less than the total force required by 150 mm of SMA to be pulled out of epoxy, the SMA wire remains at its position and does not slip during or after prestressing (while sampling preparation and after curing).

### 3.3. Gas-Gun Impact Test

**Figure 6:**
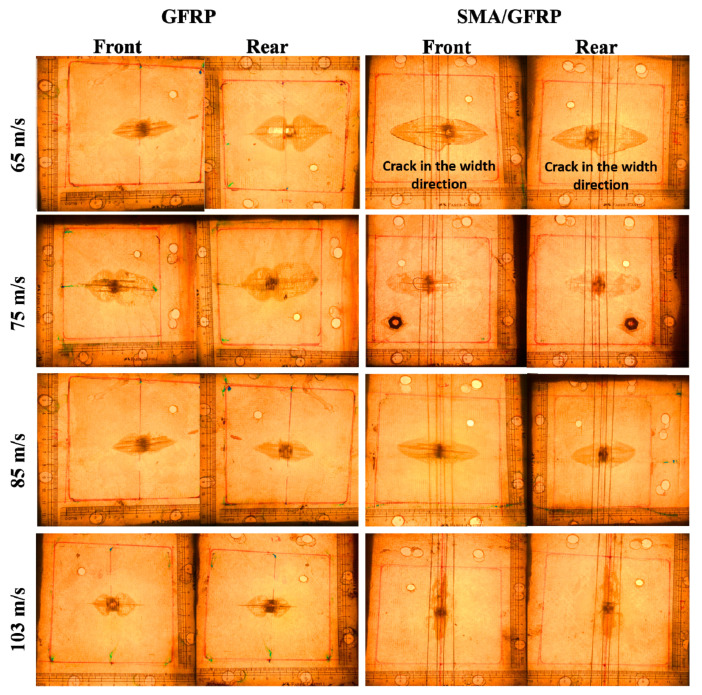
Damage visualization in GFRP and SMA/GFRP specimens (front and rear) at impact velocities 65 m/s, 75 m/s, 85 m/s and 103 m/s.

**Figure 7:**
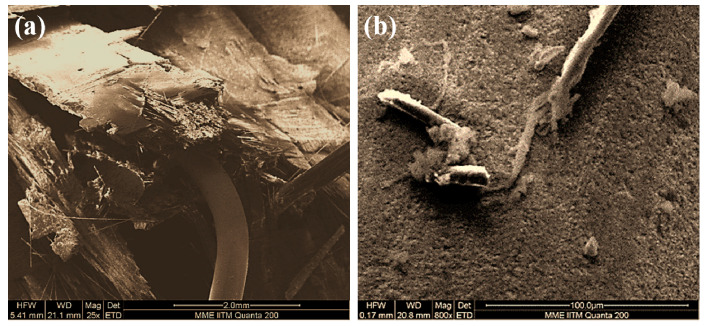
SEM micrograph of (**a**) SMA/matrix debonding failure mode, (**b**) interaction of SMA wire with GFRP and matrix, after impact.

Gas-gun impact tests were performed to calculate the energy absorbed by the GFRP and SMA/GFRP specimens via different failure modes. From Figure 6, for GFRP specimens, three major failure modes have been observed, which are primary fiber failure, matrix cracking, and delamination [33,34,35,36,37,38,39]. In contrast, energy absorbed by SMA/GFRP impact specimens has two more extra modes, namely, the SMA pull-out and the SMA deformation modes (Figure 7). At different impact velocities, the total pull-out length for the specimens are given in Table 4. Table 5 shows the comparison of residual velocities between the GFRP and SMA/GFRP specimens at different impact velocities in the medium velocity regime. The effect of the addition of SMA in slowing down the projectile is visible at all the impact velocities, especially at 75 m/s. As the velocity increases, the effect reduced, and the reasons have been explained for each velocity below.

In the primary impact region, the SMA significantly affected the damage profile of the specimen. Hence, at all different velocities, the failure modes changed as we switched from GFRP to the SMA/GFRP specimen. At 65 m/s, the total amount of energy absorbed by the GFRP and SMA/GFRP specimens was equivalent (Table 5). It is because the projectile was stuck inside the GFRP specimen at 65 m/s and was rebounded with a negligible velocity in the SMA/GFRP specimen. From the damage pattern of the SMA/GFRP specimen, it can be seen that the failure mode changed from delamination in the primary region to SMA-pull out and SMA deformation. This leads to an increase in the ballistic limit as the projectile got rebounded with the rebound velocity of 5 m/s (marked by negative sign in Table 5). Additionally, when the projectile tried to enter the SMA/GFRP laminate as it does in the GFRP laminate, it came in contact with the SMA wire. As the distance between the wires was less than the projectile diameter, the wire got pulled out from the epoxy, absorbing the energy coming from the projectile by SMA pull-out mode and SMA deformation mode. The SMA pull-out happened in the primary region and is marked by a black area, as can be seen in Figure 6. The cracks were originated from the primary region and extended in the width direction transverse to the aligned SMA.

At 75 m/s, the energy dissipation in the SMA/GFRP specimen is more than that in the GFRP specimen. Further, the total energy absorption was also the highest in comparison to all the other velocities. This was observed as the projectile slowed down tremendously. The projectile residual kinetic energy was less after passing through the specimen. From Figure 6, it can be seen that there is a small piece of GFRP, which is removed from the back face of the specimen. The length of the crack is also more in the SMA/GFRP specimen, and there is delamination in the specimen to a major extent, which is a reason behind the change in the color of the matrix in all specimens. All this can be attributed to the maximum pull-out of the SMA wire in the specimen (Table 4). As the SMA pull-out length is more, the length of the wire completing transformation strains and undergoing permanent deformation also increases. It was seen by removing the pulled-out SMA wire from the impact specimen that it was not able to return to its parent shape. Thus, the energy absorption by the SMA/GFRP specimen was maximum at 75 m/s. The volume undergoing permanent deformations increased. As seen from the damaged specimens, the black area shows the damaging behavior in the primary region. In the GFRP specimen, the primary black damaged region is more concentrated and circular because of the projectile passing through it. In SMA/GFRP specimen, this area is elliptical because of the SMA pull-out and SMA deformation. Figure 7 shows the SEM micrograph of the impacted SMA/GFRP laminate. Figure 7a shows the impact zone for the SMA-GFRP specimen and SMA/matrix debonding failure mode. It can be seen that SMA has been pulled out of the specimen leading to the crack on the back surface. The closer view of the SMA surface (Figure 7b) shows that a small amount of matrix was left on the SMA surface even after pull-out. The glass fibers are also seen attached to the surface of the SMA.

At 85 m/s, the total energy absorbed by both GFRP and SMA/GFRP specimens reduces. The projectile pierced both types of specimens. The residual velocity was more in the GFRP specimen than the SMA/GFRP specimen. As seen from Figure 6, the damaged specimens have a primary black circular region in the GFRP specimens and elliptical in the SMA/GFRP specimens, as described at 75 m/s. The extent of the damage along the wire length has been reduced, as can be seen from the specimen. The pull-out length has decreased at 85 m/s. As the length of SMA wire undergoing complete transformation has reduced, the SMA deformation energy reduces. Hence, the energy absorbed by SMA has come down.

At 103 m/s, the total energy absorption reduced down significantly in comparison to 85 m/s in both GFRP and SMA/GFRP specimens. The failure extent due to GFRP’s primary fiber failure remain almost the same because the projectile breaks the glass fibers to pass through the specimen from the other side and thus, glass fibers reach their failure strains and absorb the same amount of energy. The contact duration of the projectile with the specimen was less in comparison to the specimens at the lesser velocities. The SMA pulled out length got reduced (Table 4) significantly as the projectile ruptured the glass fibers and passed in between the SMA wires. Due to these reasons, not much dissipation happened by the SMA wire. The reduction in absorbed energy can also be understood from the damage pattern in the specimens. The damaged area in the impact specimens was the least at 103 m/s, in comparison to all other velocities in both GFRP and SMA/GFRP specimens.

### 3.4. Energy Absorbtion in Vibration

**Figure 8:**
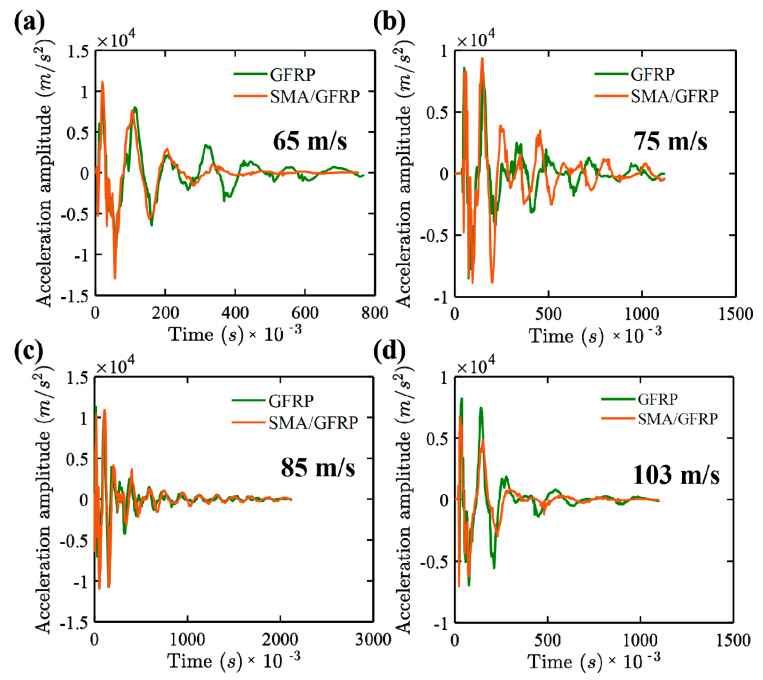
Acceleration–time response for the GFRP and SMA/GFRP specimens at different impact velocities (**a**) 65 m/s, (**b**) 75 m/s, (**c**) 85 m/s, and (**d**) 103 m/s (zoomed version of this plot is provided in Figure S1—Supplementary Materials).

**Figure 9:**
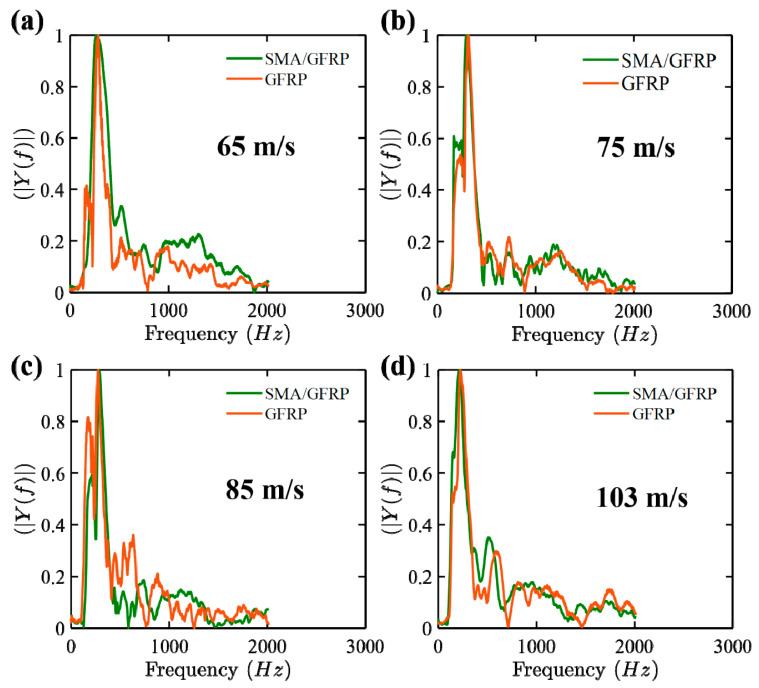
Frequency response for the GFRP and SMA/GFRP specimens at different impact velocities (**a**) 65 m/s, (**b**) 75 m/s, (**c**) 85 m/s, and (**d**) 103 m/s.

Figure 8 and Figure 9 show the acceleration–time response and frequency response plots for the GFRP and SMA/GFRP specimens when subjected to different impact velocities at 65, 75, 85, and 103 m/s, respectively. In these velocities, 65 m/s is the ballistic limit for GFRP and less than the ballistic limit for SMA/GFRP. The maximum acceleration for the specimens at different velocities is summarized in Table 6.

At all the velocities, the specimens with SMA/GFRP had fewer acceleration values in comparison to GFRP laminates. From the literature [14,32,40,41,42], it has been observed that SMA have energy dissipation capabilities and therefore also act as dampers. This may be one of the reasons of observing lower value of acceleration in SMA/GFRP specimens, which in turn could lead to higher energy dissipation by vibration in SMA/GFRP specimens. Not many changes are observed in the acceleration values for the GFRP specimens, but the range for the SMA/GFRP specimens was more.

**Figure 10:**
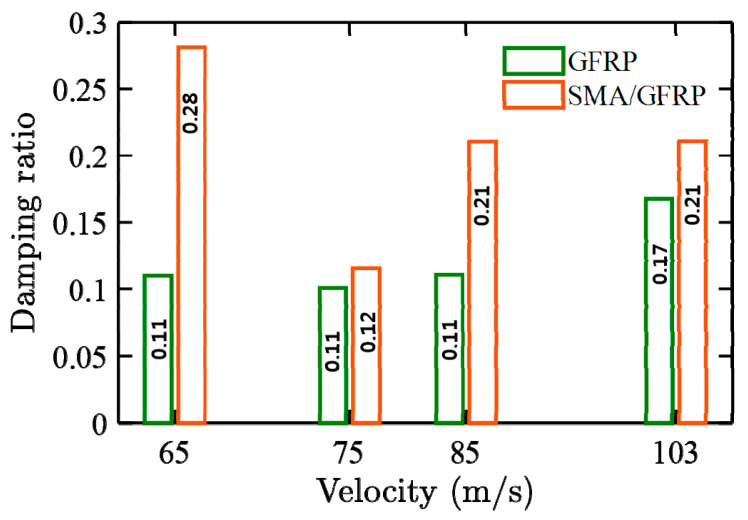
Damping ratio vs. velocity response for the GFRP and SMA/GFRP specimens at different impact velocities.

The frequency response, as shown in Figure 9, was obtained using Figure 8. From Figure 9, it can be observed that the first and second modes are the major modes observed during the impact experiments. Figure 10 shows the damping ratio vs. velocity plot for the GFRP and SMA/GFRP specimens when subjected to different impact velocities at 65, 75, 85, and 103 m/s. The damping ratios for the SMA/GFRP specimens are higher than GFRP specimens. At 65 m/s, below the ballistic limit (rebound velocity of 5 m/s), the projectile came in contact with SMA wire, which in turn absorbed energy via vibration. This may be the reason behind the high damping ratio for SMA/GFRP. However, at 75 m/s, as the SMA wire was pulled out, it was no more available to absorb energy via vibration, and thus damping ratio reduced.

At 85 and 103 m/s, the damping ratio is the same. As there was no pull-out observed at these velocities, the projectile might have come in contact with more than one SMA wire, due to which the damping ratio is high.

## 4. Conclusions

In the present work, the performance of pseudo-elastic SMA embedded composites under medium velocity impacts (65, 75, 85, and 103 m/s) was studied. Additionally, to understand the damping capabilities of the SMA embedded composites, impact experiments were conducted, and vibration signals were captured at several locations of the specimen for different impact velocities. Following this, describing the significance of the work was carried out:

1. SMA/epoxy pull-out test result signified that before SMA was pulled out from epoxy, it underwent complete phase transformation. In specific, before the overall strength degraded, SMA dominated system underwent martensitic transformations.

2. At 65 m/s, it was observed that the projectile was stuck inside the GFRP specimen, whereas it was rebounded with a small velocity of 5 m/s for SMA/GFRP specimen. Therefore, it can be concluded that embedding SMA led to an increase in the ballistic limit.

3. The effect of the addition of SMA in slowing down the projectile was visible at all the impact velocities, especially at 75 m/s. As the velocity increased (after 75 m/s), the effect was reduced. At all different velocities, the failure modes changed as we switched from GFRP to the SMA/GFRP specimen. In the SMA/GFRP specimen, the failure mode changed from delamination in the primary region to SMA-pull out and SMA deformation. This leads to an increase in the ballistic limit

4. At 75 m/s, the energy dissipation in the SMA/GFRP specimen was more than that in the GFRP specimen. Further, the total energy absorption was also the highest in comparison to all the other velocities.

5. In the GFRP specimen, the primary damage region was more concentrated and circular because of the projectile passing through it. In SMA/GFRP specimen, this area was elliptical because of the SMA pull-out and SMA deformation.

6. At 85 and 103 m/s, the total energy absorbed by both GFRP and SMA/GFRP specimens reduced. The projectile pierced both types of specimens. The residual velocity was more in the GFRP specimen than the SMA/GFRP specimen. Here, the SMA pull out length got reduced significantly as the projectile ruptured the glass fibers and passed in between the SMA wires. Due to these reasons, not much dissipation happened by the SMA wire.

7. SMA not only increased energy dissipation, but also dampened the vibrations imparted by the projectile to the specimen. The damping ratio of the SMA/GFRP specimens was higher than the GFRP specimens. The damping effect was more prominent below the ballistic limit when the projectile got rebounded (65 m/s). This can be further utilized in the systems where vibrations are the major concerns while using composites.

## Figures and Tables

**Table 1:** Material properties of pseudo-elastic (PE)-SMA.

Properties	Values (PE-SMA)	Units
Austenite Young’s modulus (E_aus_)	80	GPa
Martensite Young’s modulus (E_mar_)	40	GPa
Poisson’s ratio	0.3	-
Density	6800	Kg-m^3^
Yield stress	800	MPa

**Table 2:** Material properties of glass fibers.

Properties	Values (PE-SMA)	Units
Young’s modulus	76.6	GPa
Specific modulus	0.0340	GPa-m^3^/Kg
Specific strength	0.6200	MPa-m^3^/Kg
Poisson’s ratio	0.25	-

**Table 3:** Material properties of epoxy.

Properties	Values	Units
Specific gravity	1.28	-
Young’s modulus	3.792	GPa
Poisson’s ratio	0.30	-

**Table 4:** Total pull-out length for SMA/GFRP specimens at different impact velocities.

Impact Velocities (m/s)	Total-Pull Out Length (mm)
60	18
75	30
85	12
103	4

**Table 5:** Comparison of residual velocities for GFRP and SMA/GFRP impact specimens.

**Initial Velocity (m/s)**	65	75	85	103
**Residual Velocity (GFRP, m/s)**	0	44.2	62.2	87.2
**Residual Velocity (SMA/GFRP, m/s)**	−5	20	50.6	83.3

**Table 6:** Comparison of maximum acceleration for the GFRP and SMA/GFRP specimens at different impact velocities.

Velocity (m/s)	Maximum Acceleration (m^2^/s)	
	GFRP Specimens	SMA/GFRP Specimens
65	1.12 × 10^4^	9.4 × 10^3^
75	9.34 × 10^3^	8.5 × 10^3^
85	1.13 × 10^4^	1.09 × 10^4^
103	8.22 × 10^3^	6.71 × 10^3^

## Data Availability

The data regarding this study, either in the form of images or tables, is not available publicly.

## Supplementary Materials

The following is available online, Figure S1: Acceleration–time response for the GFRP and SMA/GFRP specimens at different impact velocities (**a**) 65 m/s, (**b**) 75 m/s, (**c**) 85 m/s, and (**d**) 103 m/s.

## Sample Availability

Samples are not available from the authors.
