# Supplementary material for: Ballistic Impact Behaviour of Glass/Epoxy Composite Laminates Embedded with Shape Memory Alloy (SMA) Wires
Source: Molecules. 2020 Dec 30;26(1):138. doi: 10.3390/molecules26010138 (PMC7796393; doi:10.3390/molecules26010138)
Supplement: Supplementary file 1 [file molecules-26-00138-s001.pdf]

molecules

*Article*

# Ballistic Impact Behavior of Glass/Epoxy Composite Laminates Embedded with Shape Memory Alloy (SMA) Wires

**Luv Verma [1], J Jefferson Andrew [1,2,*], Srinivasan M. Sivakumar [1], Gurusamy Balaganesan [3], Srikanth Vedantam [4] and Hom Nath Dhakal [5,*]**

1 Department of Applied Mechanics, IIT Madras, Chennai 600036, India
2 Department of Mechanical Engineering, Khalifa University, Abu Dhabi 127788, UAE
3 Department of Mechanical Engineering, IIT Jammu, Jammu 181 221, India
4 Department of Engineering Design, IIT Madras, Chennai 600036, India
5 School of Mechanical and Design Engineering, Anglesea Road, Anglesea Building, University of Portsmouth, Portsmouth PO1 3DJ, U.K.
* Correspondence: hom.dhakal@port.ac.uk

## Supplementary Materials

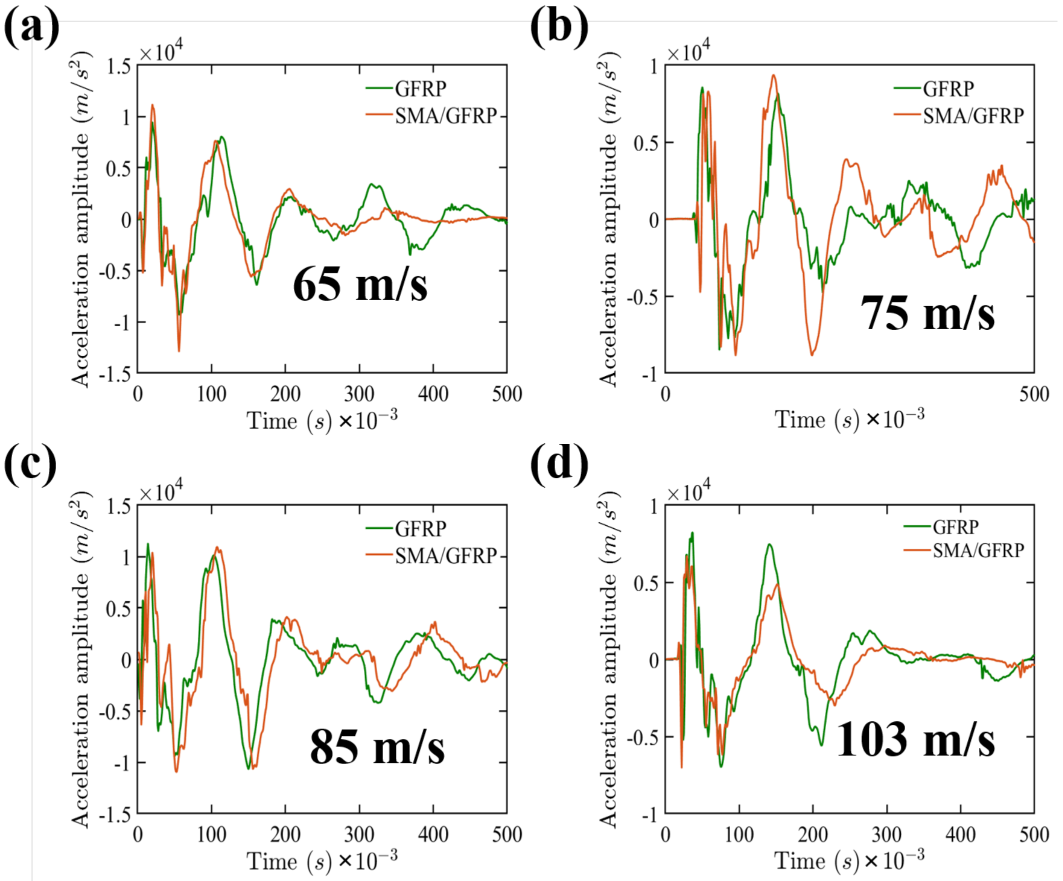


Figure S1: Acceleration-time response for the GFRP and SMA/GFRP specimens at different impact velocities (**a**) 65 m/s, (**b**) 75 m/s, (**c**) 85 m/s and (**d**) 103 m/s.
